# Sonochemical effects on fabrication, characterization and antioxidant activities of β-lactoglobulin-chlorogenic acid conjugates

**DOI:** 10.1016/j.ultsonch.2022.106240

**Published:** 2022-11-24

**Authors:** Jiayuan Liu, Gongshuai Song, Like Zhou, Yawen Yuan, Danli Wang, Tinglan Yuan, Ling Li, Guanghua He, Gongnian Xiao, Feng Chen, Jinyan Gong

**Affiliations:** a Zhejiang Provincial Key Lab for Biological and Chemical Processing Technologies of Farm Product, School of Biological and Chemical Engineering, Zhejiang University of Science and Technology, Hangzhou, Zhejiang 310023, China; b Department of Food, Nutrition and Packaging Sciences, Clemson University, SC 29634, USA

**Keywords:** LG, β-lactoglobulin, CA, chlorogenic acid, MALDI-TOF-MS, matrix-assisted laser desorption ionization time-of-flight mass spectrometry, FTIR, fourier transform infrared chromatography, CD, circular dichroism, NMR, nuclear magnetic resonance spectroscopy, DSC, differential scanning calorimetry, β- Lactoglobulin, Chlorogenic acid, Ultrasonication

## Abstract

The β-lactoglobulin-chlorogenic acid (LG-CA) conjugate was explored to be formed through ultrasonication, redox-pair method and their combination, the ultrasonication used a probe ultrasonic machine with a 6 mm probe at 270 W, and the frequency was 20–25 kHz. The formation of the conjugate was confirmed by SDS-PAGE with a larger molecular weight. Besides, Fourier infrared spectroscopy (FTIR) and Circular dichroism (CD) indicated changes in the secondary structure of the LG-CA conjugate. The α-helix and β-sheet contents of LG decreased and the unordered content increased significantly after the formation of covalent complexes. In addition, both the ultrasonic treatment and its combination with redox-pair method could significantly improve the antioxidant properties of LG. The former increased to 23.16 μmol Trolox/g sample, the latter 82–106 μmol Trolox/g sample. Therefore, ultrasonication could be used both individually and in combination with the redox-pair method to produce LG-CA conjugates with stronger antioxidant activities.

## Highlights

• The interaction between β-lactoglobulin and Chlorogenic acid by ultrasonication was studied.
• Covalent complexes were produced when β-lactoglobulin and Chlorogenic acid were sonicated at the same time.
• The highest binding content of complexes was observed with the ultrasonication/redox-pair method.
• Ultrasonication improved the antioxidant properties but reduced the thermal stability of the complex.

## Graphical abstract

**Figure:**
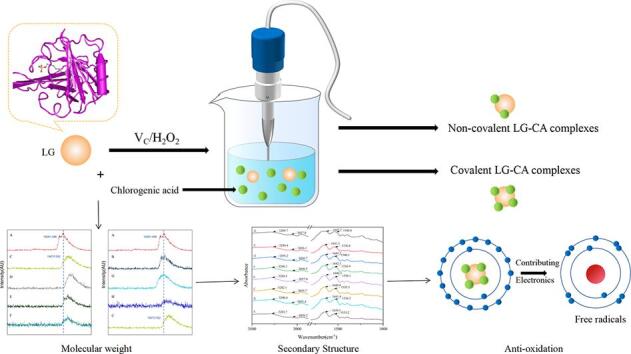

## 1 Introduction

Healthy and nutritious foods have gradually won people's hearts with the improvement of people's living standards, therefore, how to develop more innovative functional foods and improve their bioavailability has become a research focus of food industries. For example, it was found that subtly designed delivery systems could protect the biological activities of functional foods and effectively improve their solubility, physicochemical stability and bioavailability [1]. Food grade covalent complexes prepared by proteins and polyphenols are one of such kind of innovative functional foods that have better both the stability of polyphenols and the biological activities of proteins, which has been widely explored and used in the development of novel beverage or dairy products [2].

β-Lactoglobulin (β-LG, or LG) that exists in the highest content of whey protein is commonly available as a by-product of cheese processing, but it has obtained much more attention for its biological activities [3], [4]. Its monomer is composed of 162 amino acids with a molecular weight of 18,400 Da, of which the structure contains an α-helix and nine antiparallel β-sheets in a lamellar microstructure [5]. LG has been proven with good oxidation resistance, thermal stability, and other biological activities such as cholesterol reduction and antioxidant activity [5], [6], but its stability and nutritional properties are vulnerable to heating and hydrolysis [7]. However, Guo et al. [8] had found that phenolic substances could effectively improve the stability of LG structure and its biological activities. Similarly, Li et al. [9] recently reported that the covalently bound soybean isolate protein (SPI) and epigallocatechin gallate (EGCG) had much better thermal stability and stronger antioxidant properties compared to the original SPI.

Chlorogenic acid (5-*O-*caffeic quinic acid, CA) is an ester compound formed by condensation of caffeic acid and quinic acid, which is the main polyphenol in some foods such as coffee and honeysuckle [10]. Like EGCG, it has significant *in vitro* antioxidant activities against model cells relevant to the cardiovascular and cerebrovascular diseases [11], [12].

Ultrasonication is a powerful physical method that can affect the intramolecular bonds of proteins, and even disrupt their molecular structures [13], [14]. Li et al. [15] found that the ultrasonic treatment could denature the soybean prolipoprotein but with significantly improved solubility in water under certain conditions due to its cavitation and mechanical effects, which was confirmed by circular dichroism (CD) analysis for the changes of the secondary and tertiary structures of the protein. Wu et al. [16] studied the effects of ultrasonication on the physicochemical properties of whey protease, and found a higher degree of hydrolysis of whey protein after the treatment, indicating that the ultrasonic treatment could be applied to facilitate the breakdown of whey proteins for other applications, such as development of novel functional foods.

Currently, many protein–polyphenol covalent complexes have been prepared by the redox-pair method [17], by which the free radicals can be generated by the ultrasonication on proteins [9]. However, there are few studies on the relationship between ultrasonication and protein–polyphenol covalent complexes. Sun et al. investigated the structure and physicochemical properties of protein–polyphenol complexes when ultrasonication and free radical induction methods were combined, and the experiments showed that this combination significantly increased the content of protein-bound polyphenols and effectively improved the antioxidant properties of the protein [18]. A similar study was conducted by Jing et al. who used the alkaline and ultrasonication-assisted alkaline methods to prepare the egg white protein-tea polyphenol complexes [19]. However, their experiment did not sufficiently consider the different kinds of situations in which the two methods were combined. Therefore, we aimed to study the effects of ultrasonication on the formation and antioxidant activities of the LG and CA covalent complex, with the aid of modern analytical methods such as multi-spectral detection, differential scanning calorimetry and scanning electron microscopy, in order to provide insights of protein polyphenol complex for their further practical applications.

## 2 Materials and methods

### 2.1 Materials

LG (95 %, from milk) was purchased from Macklin (Shanghai, China), CA (99 %, HPLC) was purchased from Must (Chengdu, China), dialysis bag with cut-off MW of 3500D was purchased from Vake (Beijing, China), Folin phenol reagent (2 N) was purchased from Beijing Dingguo Changsheng Biotechnology Co, ltd. (Beijing, China), 2-mercaptoethanol (99 %) was purchased from Aladdin, and 2,2-diphenyl-2-picrohydrazine (97 %) was purchased from Tixiai Chemical Industry Development (Shanghai, China).

### 2.2 Preparation of protein–polyphenol complex

The radical grafting method was used according to the method described by Zheng et al. [20]. Briefly, 1.00 g LG was dissolved in 99 mL of pure water before 1 mL of 5 mol/L hydrogen peroxide solution and 0.25 g ascorbic acid were added. The mixture was stored at room temperature for 2 h before 12.40 mg CA was added and mixed. The above mixture was stored in the refrigerator (4 °C) for 24 h, then carried out by dialysis at 4 °C with interval water change in every 6 h within 48 h. Finally, the sample was placed in a freezer (-80 °C) for freezing. The samples were obtained after 24 h and dried under vacuum for 48 h.

The following 8 samples (Groups A—H) were prepared from LG with or without adding other ingredients and ultrasonic treatment for their comparison, which included:

Group A (LG): Freeze-dried LG as a control.

Group B (ULG): Freeze-dried LG after ultrasonication (270 W, 2 h).

Group C (LG-V_C_/H_2_O_2_-CA): Samples prepared according to the radical grafting procedure mentioned above using native LG.

Group D (U (LG)-V_C_/H_2_O_2_-CA): Samples prepared according to the radical grafting procedure mentioned above using sonicated LG.

Group E (U (LG-V_C_/H_2_O_2_)-CA): Based on the radical grafting procedure mentioned above, ultrasonication was applied after adding ascorbic acid and hydrogen peroxide at 270 W for 2 h, then CA was added as described to form the complex.

Group F (U (LG-V_C_/H_2_O_2_-CA)): Based on the radical grafting procedure mentioned above, ultrasonication was applied after adding CA at 270 W for 2 h to form the complex.

Group G (LG-CA): The mixture of LG and CA after standing for 24 h.

Group H (U (LG-CA)): The mixture of LG and CA treated by ultrasonication at 270 W for 2 h, and then standing for 24 h.

During the experiment, ultrasonic treatment was performed by a probe ultrasonic machine (JY92-IIDN, Ningbo Scientz Biotechnology Co., ltd., China) with a 6 mm ultrasonic probe at 270 W. The ultrasonic frequency was 20–25 kHz, the volume of the solution was 100 mL, and the liquid height (the distance from the bottom of the glass container to the ultrasonic probe) was kept at 20 mm.

### 2.3 Detection of reactive groups

The content of bound CA in the C, D, E, F, G, H samples was determined by the Folin phenol method [21]. The absorbance value of the sample was detected at 700 nm by a UV spectrophotometer. Meanwhile, CA was used as the standard curve.

The o-Phthalaldehyde (OPA) method [22] was used to determine the content of free amino groups in the samples. Taking water as the blank sample, the absorbance value of the eight groups was measured at 340 nm. The standard curve was drawn with glycine as the standard substance.

5, 5-Dithionitrobenzoic acid (DTNB) was used to detect the difference of content of free thiol groups between the samples, which was measured at 420 nm by UV spectrophotometer.

The content of free tryptophan was determined by UV spectrophotometer [23]. In detail, 0.9 mL sample (1 mg/mL) and 1 mL nitric acid were mixed in a water bath (50 °C) for 15 min. Then 4 mL of 5 mol/L NaOH and 4 mL ethanol were added respectively. The absorbance values were measured at 360 nm and 430 nm, respectively. The concentration of free tryptophan was calculated by the following formula:

C = 0.61905A_360_-0.2619A_430_.

C: Free tryptophan concentration A: Absorbance value.

### 2.4 Sodium dodecyl sulfate polyacrylamide gel electrophoresis (SDS-PAGE)

SDS-PAGE electrophoresis is the most commonly used method for protein analysis. Based on a previous reported method [24], the SDS-PAGE with minor modifications was performed, for which the thickness of the vertical plate was 1 mm, the concentration of stacking gel was 5 %, and the concentration of separating gel was 12 %. Four mL of 1 mg/mL of the eight groups was mixed with the loading buffer, in a 4:1 ratio and placed in a water bath at 100 °C for 10 min, then cooled down and centrifuged at 8000 rpm, then 10 μL supernatant was injected into the plate, and separated under the constant voltage at 200 V. After the electrophoresis, it was stained with Coomassie brilliant blue 250, and finally scanned with a Hewlett-Packard scanner (HP1000).

### 2.5 Matrix-assisted laser desorption ionization time-of-flight mass spectrometry (MALDI-TOF-MS)

Matrix-assisted laser desorption/ionization (MALDI) coupled tandem time-of-flight (TOF/TOF) mass spectrometry (MS) is an important method to identify protein covalent interactions [25]. A solution of 0.1 % trichloroacetic acid and 50 % acetonitrile was prepared separately with pure water, and then the two were mixed 1:1. The mustard acid was dissolved in the above mixed solution to make a saturated solution. After centrifugation, the supernatant was taken and dropped onto the target plate. The parameters of the mass spectrometer (BRUKER AUTOFLEX-II) were set as follows: reflection mode, excitation voltage of 200 kV, and effective flight range of 200 cm.

### 2.6 Fourier transform infrared spectroscopy (FTIR)

The potassium bromide pellet method was used to perform Fourier transform infrared spectroscopy to observe the changes in the secondary structure of the protein. The eight groups of samples were mixed with potassium bromide in a ratio of 1:100 and ground uniformly. After preparation, the sample was placed into the infrared machine (HITACHI F-4500) for scanning. Each sample was scanned three times in parallel, and potassium bromide was used as a blank control.

### 2.7 Circular dichroism (CD)

The secondary structure of the sample was detected by circular dichroism (BRIGHTTIME Chirascan). The sample was dissolved in phosphate buffer at pH 7.0 to make a final concentration at 0.2 mg/mL. The measurement conditions were as follows: room temperature at 25 °C, scanning speed at 100 nm/min, and scanning three times for each sample. The results were calculated using a Dichroweb program.

### 2.8 Endogenous fluorescence spectroscopy

The aforementioned 8 groups of samples (as listed in section 2.2) were dissolved in 10 mM phosphate buffer to prepare 1 mg/mL solutions. Using the fluorophore inside the LG molecule as a probe, the samples were scanned with a fluorescence spectrophotometer (HITACHI F-4500). The excitation wavelength was set to 280 nm, and the emission wavelength was set to 300 nm − 400 nm, and both the emission and excitation slit widths were set to 5 nm. The 10 mM phosphate buffer was used as a blank control.

### 2.9 Proton nuclear magnetic resonance spectroscopy (NMR)

The sample structure was analyzed by Bruker 400 M H NMR spectroscopy. An amount of 15 mg sample was taken into the NMR tube and mixed with enough D_2_O. Detection was performed at room temperature. The assay results were processed by MestReNova.

### 2.10 Differential scanning calorimetry (DSC)

Differential scanning calorimeter was used to measure the denaturation temperature of the samples to compare the differences in thermal stability of the eight groups of samples. Each sample (5–10 mg) was taken in a small aluminum pan, while an empty aluminum pan was used as a control, and heated from 30 °C to 180 °C. The heating rate was 10 °C /min, the nitrogen flow rate was higher than 16 mL/min. The denaturation temperature of the sample was calculated by using the TA 60 analysis software of the instrument.

### 2.11 Antioxidant analysis

Based on the previous experiments [26], three methods were used to detect the antioxidant activities of the samples: DPPH free radical scavenging experiment, ABTS free radical scavenging experiment, and FRAP reducing power experiment.

DPPH free radical scavenging rate: the sample was dissolved to make a 0.5 mg/mL solution, which was mixed with DPPH ethanol at a ratio of 1:1, reacted at room temperature for 1 h in the dark, and then measured at 517 nm, while using trolox equivalent as standard.

ABTS free radical scavenging rate: the ABTS aqueous solution (7 mmol/L) and potassium persulfate aqueous solution (2.45 mmol/L) were mixed and stored as the ready-to-use reagent in the dark at 4 °C for 12 to 16 h. This reagent was diluted with absolute ethanol, and adjusted to its OD value at 0.70 ± 0.02 measured at 747 nm. An amount of 0.5 mg/mL sample was mixed with the aforementioned adjusted ABTS working solution at a ratio of 1:3 and stood for 1 h, then measured at 734 nm, and expressed in trolox equivalents.

FRAP reduction method: all samples were dissolved in 2 % acetic acid solution to prepare their concentrations in 0.25 mg/mL. Each sample was taken by 2 mL and mixed with 1 mL of 1 % potassium ferricyanide solution, and treated in a water bath at 50 °C for 20 min before 1 mL of 10 % trichloroacetic acid solution was added. The above solution was mixed with distilled water in a ratio of 1:3, then 0.4 mL of 0.1 % ferric chloride solution was added, shaken, and allowed to stand for 5 min. The OD value of the mixture was measured at 700 nm with a microplate reader. Free radical scavenging was calculated using trolox as a standard.

### 2.12 Determination of surface hydrophobicity

Surface hydrophobicity was tested by 8-Anilino-1-naphthalenesulfonic Acid (ANS) fluorescent probe method [27], by which the eight groups of samples were measured after they were all sequentially diluted into 0.30, 0.20, 0.10, 0.05, and 0.02 mol/L solutions in 10 mmol/L phosphate buffer. At the same time, 4 mL of each sample solution was mixed with 20 μL of 8 mmol/L ANS solution, and immediately measured with a fluorescence spectrophotometer at 470 nm, while its excitation wavelength was set at 390 nm. The fluorescence intensity values vs different concentrations of the same sample are plotted, resulting in determination of the slope of the initial line segment as the surface hydrophobicity index (H_0_).

### 2.13 Statistical analysis

Each group of tests was repeated twice, and each sample was measured three times. SPSS 26.0 was used for statistical analysis, *p* value 0.05.

## 3 Results and discussion

### 3.1 Analysis of CA binding equivalents and the content of amino acid groups involved in the reaction

The ability of LG to bind CA was determined by the Folin-Ciocalteu method and expressed as total phenolic equivalents. As shown in Table 1, the CA binding equivalents in groups C to H were 75.86, 105.18, 73.80, 98.71, 55.47, 76.84 nmol/mg, respectively.

**Table 1:** Comparison of contents of CA binding equivalents, amino acid residue and antioxidant activities of LG in 8 groups with/without ultrasonication.

samples	CA (nmol/mg)	Thiol group (nmol/mg)	Free amino group (nmol/mg)	Tryptophan group (ng/mg)	DPPH scavenging activity (μmol Trolox/g sample)	ABTS + scavenging activity (μmol Trolox/g sample)	Reducing power (μmol Trolox/g sample)
A	—	46.32 ± 0.21^d^	387.34 ± 1.52 ^g^	47.99 ± 0.57^d^	54.98 ± 0.01^a^	121.80 ± 0.01^a^	22.67 ± 0.04^b^
B	—	56.52 ± 0.13^f^	395.80 ± 1.8 ^h^	56.86 ± 0.65^e^	78.14 ± 0.01^c^	127.03 ± 0.05^b^	30.00 ± 0.02^c^
C	75.86 ± 4.49^bc^	42.36 ± 0.17^b^	232.42 ± 0.59^d^	38.93 ± 0.09^a^	69.98 ± 0.01^b^	130.13 ± 0.01^b^	34.00 ± 0.05^d^
D	105.18 ± 6.37^e^	41.92 ± 0.04^a,b^	93.03 ± 0.79^a^	43.93 ± 1.12^b,c^	160.79 ± 0.01^f^	145.53 ± 0.03^c^	53.33 ± 0.07^e^
E	73.80 ± 3.66^b^	48.28 ± 0.07^e^	305.31 ± 1.28^f^	49.64 ± 0.37^d^	136.07 ± 0.01^e^	129.33 ± 0.02^b^	62.00 ± 0.08^f^
F	98.71 ± 2.28^d^	44.10 ± 0.09^c^	162.11 ± 0.98^c^	38.93 ± 0.16^a^	136.76 ± 0.09^e^	127.32 ± 0.06^b^	29.00 ± 0.07^c^
G	55.47 ± 6.68^a^	45.68 ± 0.11^a^	283.03 ± 0.79^e^	43.06 ± 0.28^b^	124.18 ± 0.04^d^	149.16 ± 0.06^d^	14.33 ± 0.03^a^
H	76.84 ± 3.16^c^	48.16 ± 0.44^e^	103.52 ± 1.14^b^	47.46 ± 0.75^c,d^	132.45 ± 0.02^e^	161.51 ± 0.02^e^	28.33 ± 0.08^c^

Note: different letters in superscript within the same row indicate significant differences among sample tests (*p* < 0.05).

(A. LG; B. ULG; C. LG-V_C_/H_2_O_2_-CA; D. U (LG)-V_C_/H_2_O_2_-CA; E. U (LG-V_C_/H_2_O_2_)-CA; F. U (LG-V_C_/H_2_O_2_-CA); G. LG-CA; H. U (LG-CA)).

Group G was prepared simply by directly mixing LG with CA resulting in the lowest bound phenolic equivalents, indicating that LG and CA could also be bound by non-covalent bonds, which was similar to the results of previous studies [28]. Group C were LG-CA covalent complexes prepared by the free radical induction method that generated hydroxyl radicals through redox reactions to attack the hydrogen atoms at hydroxyl, amino, and sulfhydryl sites on LG to form intermediates, then bound to CA [29]. Compared to group G, the CA content of group C was increased by about 20.39 nmol/mg, which indicated that the free radical induction method could bind more CA compared to the non-covalent binding [30]. The CA binding content of group H prepared under ultrasonication was 76.84 ± 3.16 nmol/mg, which was also significantly higher than that of group G. This indicated that more CA could be bound after ultrasonication on LG since the ultrasonic treatment was the only difference between these two groups (G vs H). Groups D, E, and F used both free radical induction and ultrasonication, whereas ultrasonication was applied on LG of group D, the mixture of LG and ascorbic acid/ H_2_O_2_ of group E, and the mixture of LG-CA of group F prepared by free radical induction. The levels of bound CA in these three groups were D > F > E. This indicated that the free radical induction treatment was more effective for binding more CA on the ultrasonicated LG.

In the OPA method to measure free amino content, 1 % SDS solution, which is an anionic surfactant that can break hydrogen bonds, was added to bind the hydrophobic part of LG. Mercaptoethanol allows the reduction of the disulfide bond of LG, which facilitates the binding of SDS and LG. Therefore, the addition of SDS and mercaptoethanol could disrupt the non-covalent bonds, proving that the decrease of free amino content was the result of covalent bonding [31]. As shown in Table 1, compared with LG (387.34 ± 1.52 nmol/mg), the content of free amino in the LG-CA complex (232.42 ± 0.59 nmol/mg) prepared by the free radical induction method was significantly decreased in group C, which indicated that the free radical induction method had effectively decreased the free amino groups in LG-CA covalent complexes. However, in comparison of LG-CA after ultrasonication, the free amino group content of LG significantly increased to (395.8 ± 1.8 nmol/mg), which was ascribed to the LG structure opened by the ultrasonication, leading to the hidden free amino groups exposed. The contents of free amino groups in the three groups D, E, and F ranged from high to low were: E (305.31 ± 1.28 nmol/mg > F (162.11 ± 0.98 nmol/mg) > D (93.03 ± 0.79 nmol/mg). Although all three groups of samples were prepared by the combination of the free radical induction method and ultrasonication, their significant difference in the content of free amino groups could be found. This might be caused by ultrasonication on the different materials. In group D, ultrasonication was applied to LG solution, which significantly changed the structure of LG, exposing the binding sites inside LG to bind more CA. Group E, ultrasonication was applied to the mixed solution of LG and redox agent. Ultrasonication might disrupt the redox process of the free radical induction method, thus affecting the covalent binding of LG and CA. For group F, ultrasonication was applied to the LG-CA complex, and the ultrasonication at this time may play a certain degree of the destructive effect on the LG-CA complex, resulting in a decrease in its binding content. Meanwhile, we found a significant decrease in the free amino content in the group H compared to group B of approximately 292.28 nmol/mg. This suggested the presence of covalently bound LG-CA complexes at this time. This might be attributed to –OH radicals generated by the action of ultrasonication on water molecules, which can also attack the hydrogen atoms of the LG group, forming intermediates that can covalently bind to CA [32].

Table 1 shows both the free thiol groups and free tryptophan contents of 8 groups. The same trend of variation as that of free amino groups was obtained. This proved that C—N and C—S bonds could be formed between the phenolic ring of CA and the nucleophilic group of LG when LG was bound to CA, which was also consistent with the conclusion drawn by Li et al. [33].

### 3.2 Formation of covalent bonds

**Fig. 1:**
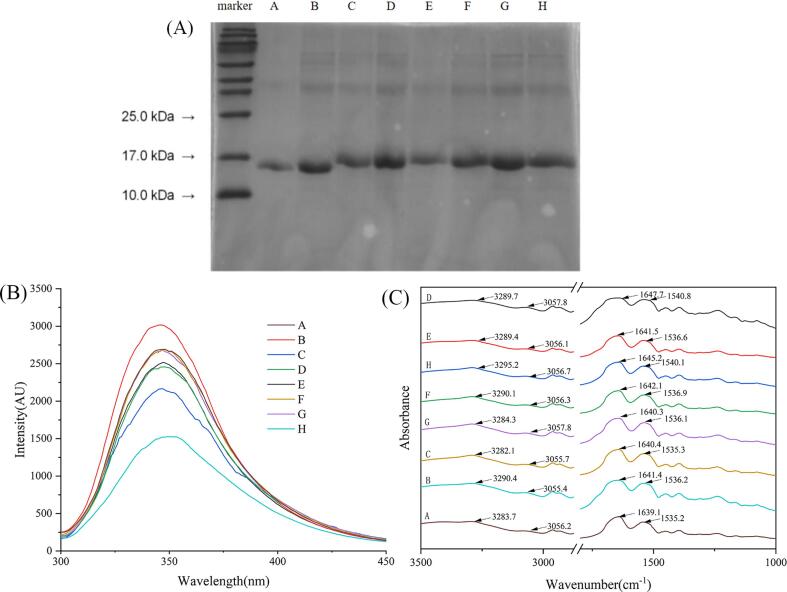
(A) SDS-PAGE of different groups of samples. (B) Endogenous fluorescence spectra of different groups of samples. (C) Fourier transformed infrared (FTIR) of different groups of samples. (A. LG, B. ULG, C. LG-V_C_/H_2_O_2_-CA, D. U (LG)-V_C_/H_2_O_2_-CA, E. U (LG-V_C_/H_2_O_2_)-CA, F. U (LG-V_C_/H_2_O_2_-CA), G. LG-CA, H. U (LG-CA)).

As shown in Fig. 1**A**, this experiment was carried out to verify the generation of covalent complexes by SDS-PAGE for the samples of groups C, D, E, F, H. Original LG of whey protein in group A had a smaller molecular weight of 10–20 kDa [24]. In comparison, there was no difference in the band positions of the samples of groups A, B and G. This indicated that the ultrasonication did not change the molecular weight of LG in samples of groups A, B, and G, and the physical mixing of LG and CA did not change the molecular weight of LG either. In contrast, the electrophoresis bands of groups C, D, E, F, and H all appeared at higher molecular weight positions than group A. Since SDS only broke non-covalent bonds but not covalent bonds [32], the bands shown with high molecular mass in SDS-PAGE indicated the stability of the LG-CA complexes. This also indicated that the CA in the complexes of groups C, D, E, F, and H were covalently attached, but the delicate difference of their molecular weights could not be accurately determined by SDS-PAGE. It is worthy of mention that the complexes of groups D, E, F were prepared by ultrasonication/redox-pair method, and group C was prepared by the redox method. The covalent binding of lactoferrin (LF) and polyphenols was also reported [29]. In that study, the protein–polyphenol covalent complexes could be effectively prepared by the redox method, resulting in the band position of complexes moving into positions with higher molecular mass on SDS-PAGE than that of LF. The band position of the group H was also slightly shifted, which indirectly proved our previous hypothesis that ultrasonication could also generate hydroxyl radicals that attacked the nucleophilic group of LG and bind to CA with covalent bonds [34].

**Fig. 2:**
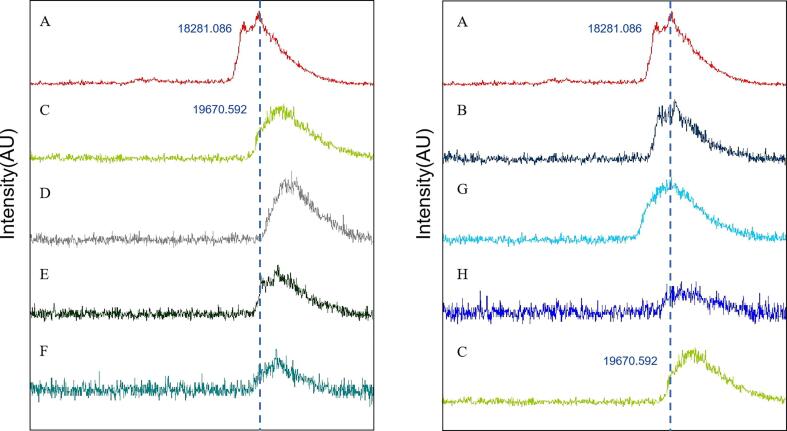
MALDI-TOF-MS of LG and LG-CA complexes. (A. LG, B. ULG, C. LG-V_C_/H_2_O_2_-CA, D. U (LG)-V_C_/H_2_O_2_-CA, E. U (LG-V_C_/H_2_O_2_)-CA, F. U (LG-V_C_/H_2_O_2_-CA), G. LG-CA, H. U (LG-CA)).

The molecular weight of LG and LG-CA complexes was analyzed by MALDI-TOF-MS. As shown in Fig. 2, in this condition, the molecular weight of LG was 18281 Da, which was consistent with the reported results [35]. As can be seen, the peak positions of groups B and G did not change compared to group A, indicating that neither the ultrasonication nor the physical mixing of CA changed the molecular weight of LG. This was mainly due to the weak bonding force in non-covalent mixing, the excitation voltage of the mass spectrometer and the separation of the electromagnetic field could break the non-covalent bond between LG and CA. However, the covalent bonds were stronger and could not be easily broken. Therefore, the increase in molecular weight could indicate the formation of covalent bonds between LG and CA [36]. It was very obvious that the positions of the peaks of the free radical-induced covalent complexes (group C), the groups with combined ultrasonication/redox-pair method (groups D, E and F) and the ultrasonication LG-CA group (group H) were significantly shifted to the right. It indicated that LG-CA covalent complexes were present in the five groups of samples. Among them, the molecular weight of group C was 19,671 Da and that of CA was 354.3 Da, indicating that there might be four CA molecules bound to LG when LG and CA were covalently combined.

### 3.3 Changes in secondary structure

As shown in Fig. 1**B**, fluorescence intensity of the LG of group B, compared to the control group A, increased significantly after the ultrasonication, which was ascribed to the changes of the structure of LG induced by the ultrasonic treatment, leading to more chemicals such as tryptophan and tyrosine inside LG to be exposed. The result was in agreement with the report of Cheng et al. [37] Compared with LG, the intensity of the fluorescent peak of LG with the addition of CA reduced. This result might be due to the participation of fluorophores such as tryptophan in LG in the formation process of LG-CA complex, which led to fluorescence quenching. The covalent bond in LG-CA in group C resulted in a remarkable decrease in the fluorescent peak intensity than in the physical mixture of LG-CA in group G. The lowest fluorescence intensity was observed in group H, which had a significant red shift in its spectrum, indicating that the LG unfolded at this time and the secondary structure underwent irreversible changes. This also indicated that the covalent binding was stronger and more stable, and the CA binding rate was higher, thus leading to a more significant change in the fluorescence spectrum.

As shown in Table 2, based on the CD analysis, the secondary structure of original LG was composed of 11.3 % α-helix, 39.9 % β-sheet, 21.8 % β-turn, and 27 % random coil. After the ultrasonication, the secondary structure of LG changed significantly, leading to the contents of α-helix and β-sheet of group B decreasing to 10.7 % and 32.4 %, respectively, and the content of random coil increasing to 35.2 %, which indicated that the structure of LG was partially unfolded, or loosely broken apart. The content of random coil of the covalent complexes LG-CA in group C was increased by about 6 %, indicating that the combination of LG and CA deconstruct the secondary structure of LG when the covalent complexes were prepared by free radical induction method. This might be because the free –OH attacked the hydrogen bond of LG, which changed the arrangement of the LG secondary structure. The contents of α-helix, β-sheet, β-turn and random coil in the group G were not different from those in the group A, indicating that CA did not affect the secondary structure of LG when LG was physically mixed with CA. The random coil content increased to 35.5 %, 42.2 %, and 44.4 % in the samples of groups D, E, and F, respectively. This indicated that the secondary structure of LG also changed significantly in the three groups complexes, and the degree of change was higher than that of group B. This was the result of the simultaneous action of ultrasonication and free radical induction method. Compared to the group B, the random coil content of group H increased by nearly 3 %, which indicated that the addition of CA affected the secondary structure of LG under ultrasonic conditions.

**Table 2:** Comparison of oxidation resistance, surface hydrophobicity and thermal stability of LG in 8 groups with/without ultrasonication.

Samples	α-Helix (%)	β-Sheet (%)	β-Turn (%)	Random coil (%)	H_0_ (slope × 10^2^)	Ts (℃)	ΔH (mJ/mg protein)
CA	–	–	–	–	–	88.8	6.6
A	11.3	39.9	21.8	27.0	3257.6	97.2	206.8
B	10.7	32.4	21.7	35.2	2996.1	81.1	233.1
C	12.2	33.3	21.4	33.0	2626.6	99.3	197.6
D	10.6	28.7	25.1	35.5	1512.3	85.2	220.6
E	10.3	27.3	20.1	42.2	1966.7	136.5	209.1
F	11.4	25.1	19.1	44.4	2017.8	78.7	213.3
G	11.9	39.4	22.2	26.5	2760.2	131.1	199.2
H	12.1	27.5	22.3	38.0	2733.0	84.7	208.9

(A.LG; B. ULG; C. LG-V_C_/H_2_O_2_-CA; D. U (LG)-V_C_/H_2_O_2_-CA; E. U (LG-V_C_/H_2_O_2_)-CA; F. U (LG-V_C_/H_2_O_2_-CA); G. LG-CA; H. U (LG-CA)).

According to the result of FTIR (Fig. 1**C)**, the main peaks of LG spectra at were 3283.7 cm^−1^ (amide A band), 1639.1 cm^−1^ (amide I band) and 1535.2 cm^−1^ (amide II band), representing N—H stretching, C—O stretching and C—N stretching, respectively. There was a significant blue shift in the spectrum of LG after ultrasonication, and the peak of the amide A band of LG in group B was blue-shifted by 6.7 nm, indicating that ultrasonication had an effect on the change of the secondary structure of LG. After the covalent binding of LG and CA, the amide A band of LG also shifted, for example, its IR spectrum peak of LG in group C had a red-shift by 1 ∼ 2 nm, indicating that the N—H stretching in the amide A band has changed and –NH_2_ was involved in the reaction of LG-CA bound, which was responsible for the corresponding changes in the secondary structure of LG. The band of group G showed almost no change compared to that of group A, indicating that the simple physical mixing of CA did not lead to changes in the secondary structure of LG. The band of group H showed a blue shift compared to that of group B, for which the peaks of the spectra were shifted to 3295.2 cm^−1^, 1645.2 cm^−1^, and 1540.1 cm^−1^, from 3290.4 cm^−1^, 1641.4 cm^−1^, and 1536.2 cm^−1^, respectively, indicating that other factors besides the factor of ultrasonication might also be responsible for the change in LG structure at this time.

**Fig. 3:**
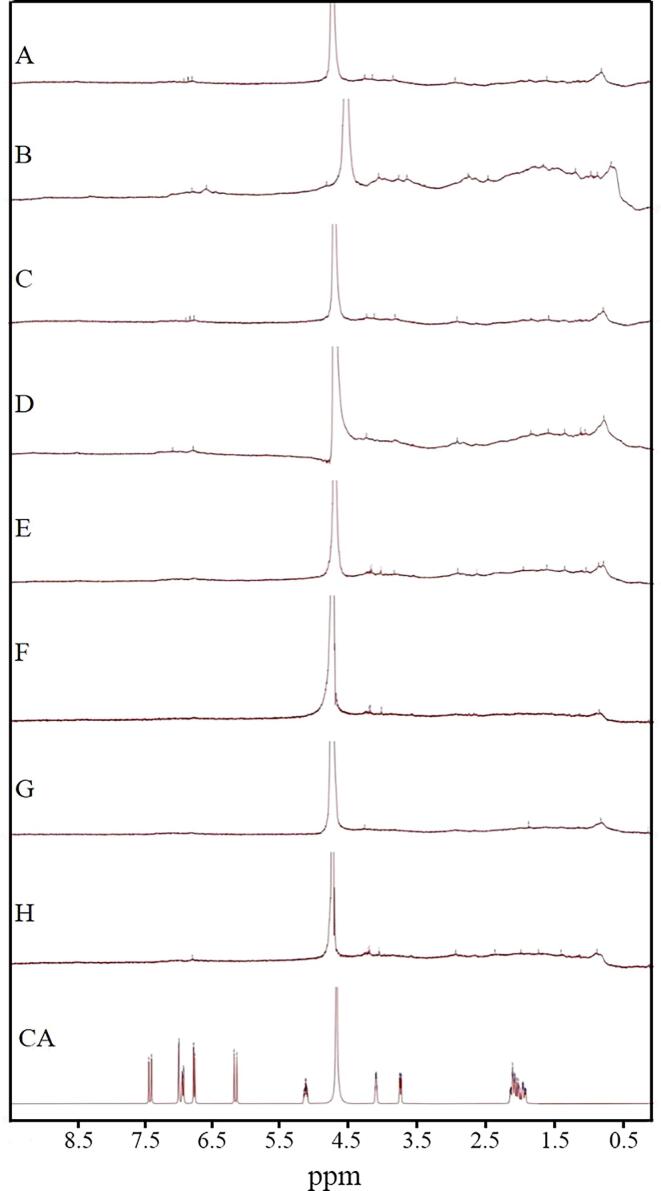
NMR spectra of different groups of samples. (A. LG, B. ULG, C. LG-V_C_/H_2_O_2_-CA, D. U (LG)-V_C_/H_2_O_2_-CA, E. U (LG-V_C_/H_2_O_2_)-CA, F. U (LG-V_C_/H_2_O_2_-CA), G. LG-CA, H. U (LG-CA)).

NMR is an important technical tool that can analyze protein structure with high resolution. As seen in Fig. 3, the groups C, D, E, F, G, H all showed relatively broad ^1^H signals, which indicated that CA had an effect on the mobility of the LG molecule. Meanwhile, the spectra of the LG-CA complexes all showed some similarity with LG of group A, which indicated that those complexes were derivatives of LG. The ^1^H NMR spectra of LG in group B showed new peaks in 6.55 ppm, 2.71 ppm, 0.81 ppm, which was due to the ultrasonication that disrupted the structure of LG and exposed the internal hidden tyrosine and tryptophan moieties [38]. In the physical mixture of LG-CA in group G, the ^1^H NMR spectrum of LG-CA was not much different from that of group A, and no characteristic peak of CA appeared. In contrast, groups D, E, F, H showed characteristic peaks of CA by covalent binding (at 1.5–2 ppm). The relative intensities of the peaks of these four groups of samples changed, indicating that the secondary structure of LG changed significantly upon covalent binding of LG and CA.

### 3.4 Analysis of surface hydrophobicity

The ANS method is an effective method to measure the surface hydrophobicity of proteins. As shown in Table 2, the surface hydrophobicity index (H_0_) of LG in group B, compared to LG in group A, decreased to 2996.1 after the ultrasonication indicating that ultrasonication effectively improved the hydrophilicity of LG. It was probably due to the conformational rearrangement of LG and the exposure of its internal hydrophilic groups. Similarly, the H_0_ of LG in group C was reduced to 2626.6 after covalent binding of CA. Ming et al. [39] found that the incorporation of tea polyphenols (TP) into whey protein isolate (WPI) significantly reduced the H_0_ of WPI, because the addition of polyphenols changed the net charge of protein molecules, increasing the hydrophilicity of the protein. The H_0_ values of the samples in groups D, E, F, H were ranked from low to high in the following order, D < E < F < H (1512.3 < 1966.7 < 2017.8 < 2733), all significantly lower than LG (3257.6) in group A. This was ascribed to the following two reasons: on the one hand, the cavitation effect of ultrasonic treatment unfolded the LG structure and exposed some internal polar amino acids (such as tyrosine, etc.), which increased the hydrophilicity of LG itself; on the other hand, ultrasonication exposed more binding sites such as tryptophan and more CA bound to hydrophobic amino acids. Thus, the binding of CA increased the hydrophilicity of LG and decreases the surface hydrophobicity index.

### 3.5 Analysis of thermal stability and oxidation resistance

Currently, DSC have been developed to study the denaturation of individual proteins at different temperature ranges. As listed in Table 2**,** the value of transition temperature (Ts) for LG in group A was 97.2 °C, which indicated that LG had lost its hydration layer and had started to denaturation at this time. The Ts in group B was 81.1 °C, with significantly lower values. This was due to the fact that the mechanical effect of ultrasonication promoted the unfolding of the protein structure, which reduced the thermal stability of LG. This was similar to the conclusion obtained by Sun et al. [40], the mechanical effect of high ultrasonic power can destroy the structure of protein and reduce its thermal stability. However, the Ts values of LG increased by 2.1 °C in group C and 33.9 °C in group G, indicating that the thermal stability of LG was effectively improved by binding CA. Similar results were obtained by Ming et al. [39].

By comparing the enthalpy change values of LG in different samples, it was found that the ΔH values of LG in groups C and G decreased by 92.0 mJ/mg protein and 7.6 mJ/mg protein, respectively. This indicated that less energy was required to open the internal structure of LG at this time, which was due to the decreased portion in ordered structure and increased portion in unordered structure of LG after binding CA. Similar findings were found by Ferraro et al. [41] in their study of Rosmarinic acid (RA) and LG interaction, where the ΔH values decreased significantly when LG bound RA, indicating that the aggregates of LG and RA were more stable than those of LG. Compared with ΔH of LG in group A, the ΔH values increased by 26.3 mJ/mg protein, 13.8 mJ/mg protein, 2.3 mJ/mg protein, and 6.5 mJ/mg protein in groups B, D, E, F, and H, respectively, which might be due to the prolongation of ultrasonication time and the aggregation of proteins [42].

Antioxidant activities of samples determined by the three aforementioned methods were listed in Table 1. The DPPH free radical scavenging capability of group A was equivalent to 54.98 μmol Trolox/g, after ultrasonication, the antioxidant activity of LG in group B was increased to 78.14 μmol Trolox/g, indicating that ultrasonic action improved the antioxidant activity of LG. The value of the ability to scavenge DPPH of Group C increased by 15 μmol Trolox/g sample, showing that the combination of CA could also effectively improve the antioxidant activity of LG, this might be due to the fact that the polyphenol conjugation contributed more –OH after binding to LG. This phenomenon was similar to the result of the interaction of TP and WPI [43]. The DPPH radical clearing capacity of samples treated by the ultrasonication/redox-pair method in three groups (i.e., groups D, E and F) increased by about 66–90 μmol Trolox/g compared to that of group C. This was partly due to the higher CA binding content of these three groups of samples compared to group C. On the other hand, it had been found that ultrasonication and redox-pair method, when acting simultaneously, might cause hydrolysis of proteins and produce various bioactive peptides with high oxidative activity [32]. In addition, as the results of Wei et al. the experiments revealed a higher scavenging capacity of DPPH radicals in the group G than in the group C, suggesting that non-covalently bound polyphenols had higher radical scavenging activity when the same mass of LG was covalently bound to polyphenols [36]. This might be due to the covalent binding involvement of amino acid residues such as tryptophan, which affected the free radical scavenging effect [44].

Similar results were obtained in the ABTS radical scavenging assay and the FRAP assay. For example, the ability of LG to scavenge ABTS radicals after ultrasonication in group B increased by about 5.23 μmol Trolox/g compared to LG in group A. The ability of LG-CA to scavenge ABTS after CA addition was higher in all six groups than in groups A and B, and from highest to lowest: group H > group G > group D > group C > group E > group F (161.51 μmol Trolox/g > 149.16 μmol Trolox/g > 145.53 μmol Trolox/g > 130.13 μmol Trolox/g > 129.33 μmol Trolox/g > 127.32 μmol Trolox/g). This also indicated that both ultrasonication and CA addition could effectively improve the antioxidant properties of LG. The difference was that the non-covalently bound group G had a weaker Fe^3+^ reduction ability although its free radical scavenging ability was higher than that of the group C (the reducing power of LG-CA in group C was about 19.67 μmol Trolox/g higher than that of LG-CA in group G). It indicated that the LG-CA complexes prepared by the free radical induction method had a better ability to reduce Fe^3+^ than the LG-CA complexes prepared by physical mixing [45].

### 3.6 Changes in microstructure

**Fig. 4:**
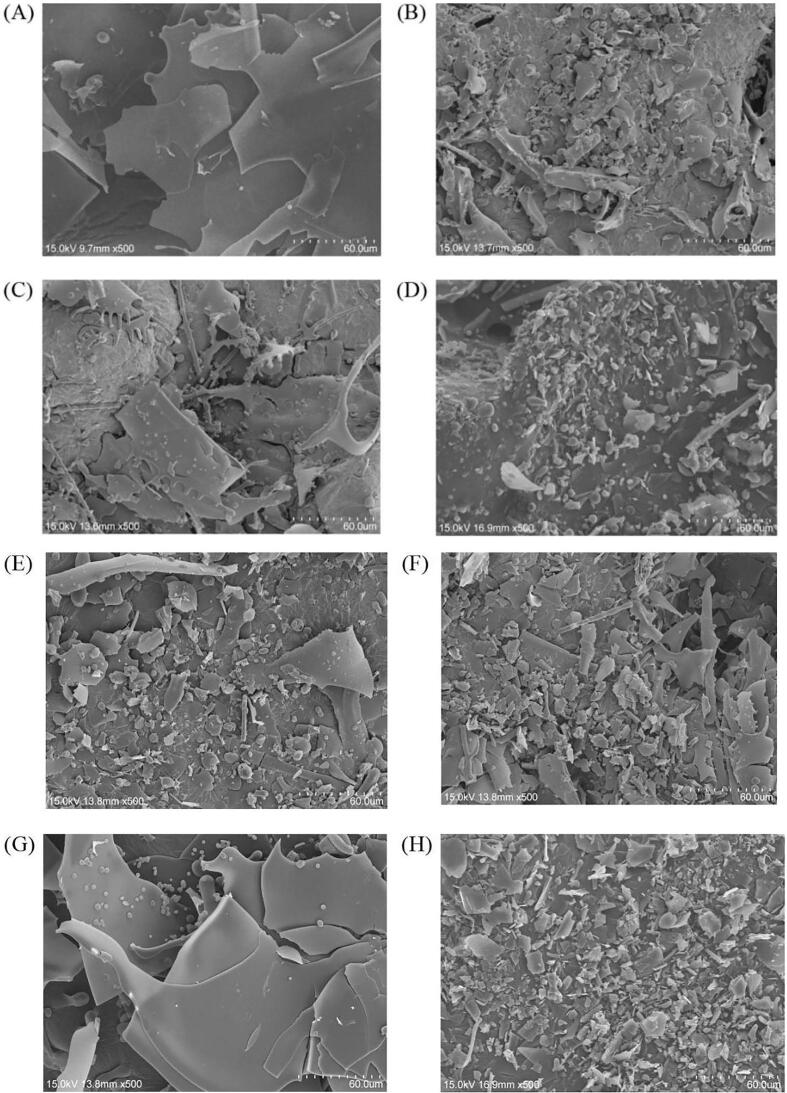
Scanning electron microscopy (SEM) of different groups of samples. (A. LG, B. ULG, C. LG-V_C_/H_2_O_2_-CA, D. U (LG)-V_C_/H_2_O_2_-CA, E. U (LG-V_C_/H_2_O_2_)-CA, F. U (LG-V_C_/H_2_O_2_-CA), G. LG-CA, H. U (LG-CA)).

The microstructure of LG in the different samples was observed by scanning electron microscopy (SEM). As shown in Fig. 4**A**, LG showed a regular sheet structure below 60 μm after vacuum drying. In comparison, after the ultrasonication, the structure was obviously destroyed. The large-area sheet in LG disappeared, instead, a large number of uniform small fragments appeared (Fig. 4**B**). Similar phenomenon was also reported in the SEM images of rapeseed protein isolates (RPI) after ultrasonic treatment, which was subject to the formation of local hot spots after the bubble collapse in the medium induced by ultrasonication, as well as the shear force generated by microfluidics and shock waves [46]. The edges of the fragments of the sample (group B) were smooth and closed to each other, indicating that the long time ultrasonication made the loose structure of LG to aggregate together, and the macromolecular proteins were reorganized to form a new aggregated structure. In Fig. 4**C**, when CA and LG were covalently bound together, many new needle-like or rod-like structures, and small particles appeared simultaneously, probably because the covalent binding weakened the effect of intramolecular and intermolecular hydrogen bonds. Similar rod-like structures could be found in groups D, E, F and H that also had covalently bounded LG-CA complexes.

## 4 Conclusions

The free radical induction method is an effective method for binding protein and polyphenols. As a result, the covalent bond between LG and CA changed the secondary structure of the original LG, and was more stable than that with non-covalent bonds. The formed covalent complex can effectively improve the physical and chemical properties of LG, such as thermal stability and antioxidant activity, etc. Likewise, the combination of CA and LG can improve the thermal stability and oxidation resistance of LG.

Ultrasonication is an effective method for changing the structure of protein. Based on SEM, it was found that the structure of LG was significantly damaged by ultrasonic treatment, for instance, the original spherical structure was broken into pieces, and the structure was obviously loose. This was also obvious in the determination of secondary structure. The secondary structure of LG in 8 groups was calculated by analytical means such as circular dichroism, and the content of LG irregular curls after ultrasonic treatment was significantly increased. Tryptophan is one of the key amino acids in the binding process of LG and CA, of which the content in the samples after the ultrasonic treatment was significantly increased. The only drawback of the ultrasonication was that ultrasonication caused a certain degree of damage to the thermal stability of LG. Long-time ultrasonication may cause protein aggregation and change its structure, thereby reducing thermal resistance.

In conclusion, our study showed that LG and CA can combine to form covalent complexes by free radical induction, and the formation of covalent complexes can be promoted by ultrasonication. LG combined with CA can effectively improve its antioxidant and hydrophilic properties.

## Funding

This work was financially supported by the National Natural Science Foundation of China (No. 31871763), the Major Agriculture and Social Development Projects of Hangzhou (2022ZDSJ0206), Qianjiang Special Expert Project of Hangzhou.

## CRediT authorship contribution statement

**Jiayuan Liu:** Investigation, Writing – original draft. **Gongshuai Song:** Data curation, Methodology. **Like Zhou:** Writing – review & editing. **Yawen Yuan:** Validation, Data curation. **Danli Wang:** Visualization, Investigation. **Tinglan Yuan:** Formal analysis, Methodology. **Ling Li:** Formal analysis, Software. **Guanghua He:** Resources, Conceptualization. **Gongnian Xiao:** Resources, Conceptualization. **Feng Chen:** Conceptualization. **Jinyan Gong:** Supervision, Funding acquisition.

## Declaration of Competing Interest

The authors declare that they have no known competing financial interests or personal relationships that could have appeared to influence the work reported in this paper.

## Data Availability

The data that has been used is confidential.
